# Application of artificial intelligence and machine learning in lung transplantation: a comprehensive review

**DOI:** 10.3389/fdgth.2025.1583490

**Published:** 2025-05-01

**Authors:** Xiting Liu, Wenqian Chen, Wenwen Du, Pengmei Li, Xiaoxing Wang

**Affiliations:** ^1^ Department of Pharmacy, China-Japan Friendship Hospital, Beijing, China; ^2^ Department of Pharmacy Administration, Clinical Pharmacy School of Pharmaceutical Sciences, Peking University, Beijing, China

**Keywords:** lung transplantation, artificial intelligence, machine learning, organ allocation, prognosis

## Abstract

Lung transplantation (LTx) is an effective method for treating end-stage lung disease. The management of lung transplant recipients is a complex, multi-stage process that involves preoperative, intraoperative, and postoperative phases, integrating multidimensional data such as demographics, clinical data, pathology, imaging, and omics. Artificial intelligence (AI) and machine learning (ML) excel in handling such complex data and contribute to preoperative assessment and postoperative management of LTx, including the optimization of organ allocation, assessment of donor suitability, prediction of patient and graft survival, evaluation of quality of life, and early identification of complications, thereby enhancing the personalization of clinical decision-making. However, these technologies face numerous challenges in real-world clinical applications, such as the quality and reliability of datasets, model interpretability, physicians' trust in the technology, and legal and ethical issues. These problems require further research and resolution so that AI and ML can more effectively enhance the success rate of LTx and improve patients' quality of life.

## 1 Introduction

Lung transplantation (LTx) is primarily utilized for the treatment of chronic end-stage lung diseases. When a patient with chronic end-stage lung disease exhibits progressive deterioration in lung function despite receiving optimal treatment, and no further medical or surgical interventions are feasible, with a mortality risk exceeding 50% within two years, LTx should be considered (1).

Artificial intelligence (AI) investigates the use of computer systems to simulate human cognitive processes and intelligent behaviors, such as learning, reasoning, self-correction, and environmental perception, with the goal of achieving higher functionality. Machine learning (ML), a branch of AI, focuses on developing algorithms that autonomously learn from data and recognize patterns. By training on input datasets, these algorithms discern fundamental principles, which are then used to make informed decisions or predictions, thereby enhancing system intelligence and adaptability (2).

**Figure 1:**
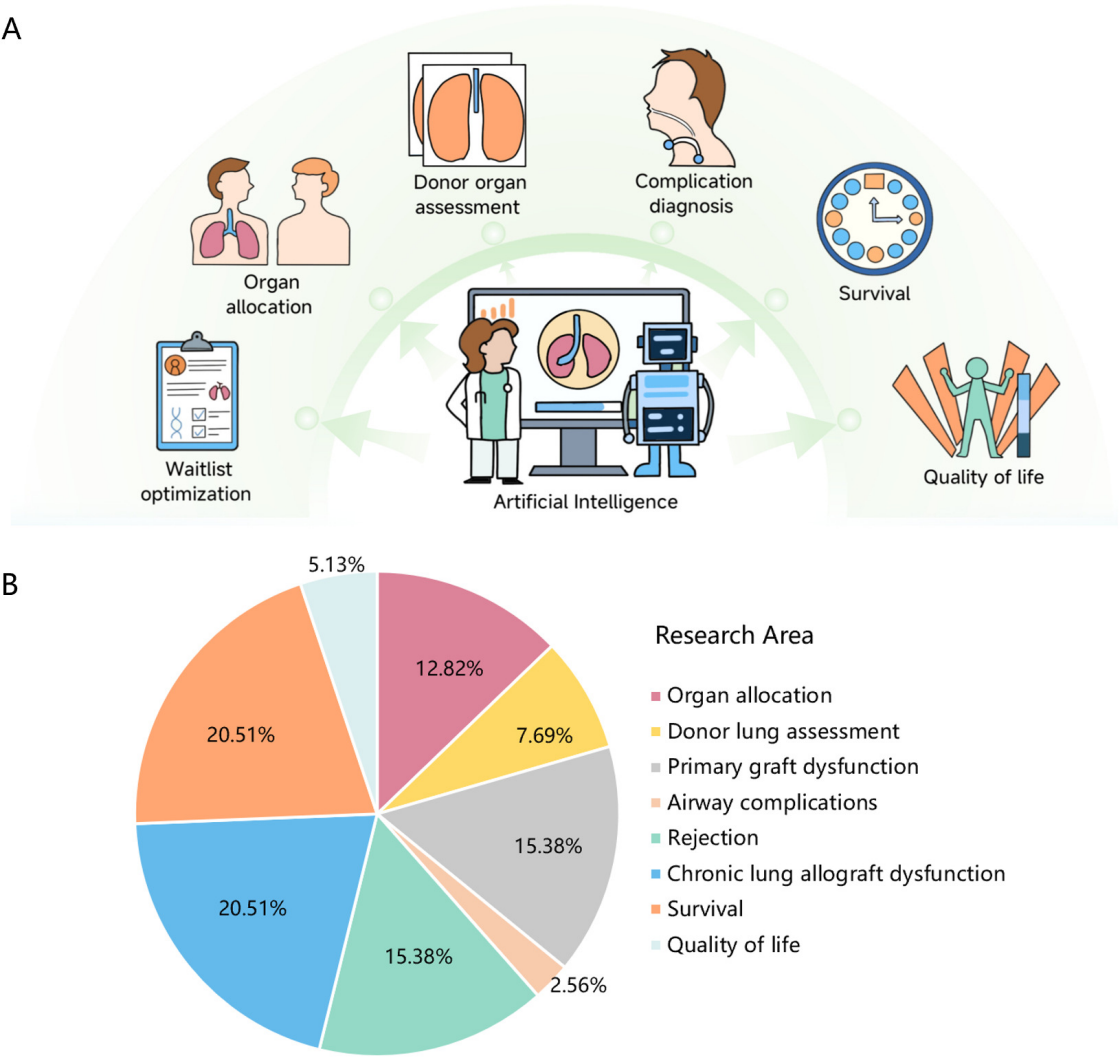
Artificial intelligence in lung transplantation: current applications and research areas.

In recent years, the application of AI and ML in medicine has expanded significantly, encompassing disease risk prediction, diagnosis, and treatment, thus improving clinical diagnosis, treatment, and management levels. In the field of LTx, researchers can employ ML as a decision support tool for various aspects, including waitlist optimization, organ allocation, donor organ assessment, postoperative complication diagnosis (e.g., primary graft dysfunction (PGD), airway complications), clinical outcome prediction (e.g., survival, quality of life), and long-term monitoring [e.g., rejection, chronic lung allograft dysfunction (CLAD)] (Figure 1A). Figure 1B illustrates the distribution of research focus areas in LTx, quantified by their relative prevalence in recent studies. CLAD and survival (each account for 20.51%) represent the most actively investigated topics, while AI applications in quality of life (5.13%) and airway complications prediction (2.56%) remain emerging areas requiring further exploration. This review provides a comprehensive summary of studies utilizing ML techniques in LTx, highlighting key advancements, current limitations, and potential avenues for future research.

## 2 Pre-transplant

### 2.1 Organ allocation

Predicting risks and outcomes by considering various factors and constraints is essential in medical practice, especially in solid organ transplantation, where precise predictive models are critical for identifying patients with the greatest need. Given the persistent organ shortage, these models play a key role in balancing waitlist mortality and post-transplant survival, ensuring efficient and equitable organ allocation (Table 1) (3).

**Table 1:** Machine learning in graft allocation and donor organ assessment.

Reference	Objective	Dataset description	Dataset split (train/test)	Predictors	ML method/model	Evaluation of best model/Model performance	Top predictive variables
Mark et al. (4)	Prognosis: 5-year survival	IRD: 1010; non-IRD: 12013; WL: 19217; UNOS dataset	Cross-validation: 80%/20%	Clinical and laboratory variables	The Cox proportional hazards model and the RSF model	Root mean square error: 5.3	None reported
Dueñas-Jurado et al. (5)	Prognosis: 6-month survival	404 lung transplants; single center	None reported	36 donor-recipient pairing characteristic input variables	The optimal Logistic Regression using Initial Covariates and Product Units model	None reported	Positive variables: higher pre-transplant and post-transplant functional vital capacity Negative variables: low FEV1 pre-transplant
Zafar et al. (6)	Prognosis: 1-, 5-, 10-year survival, and half-life prediction	15214 double lung transplants, UNOS	Single split: 70%/30%	Recipient-, donor-, and transplant-related variables	Cox-LASSO regression model	C statistics: 1 year: 0.67 5 year: 0.64 10 year: 0.72	None reported
Brahmbhatt et al. (7)	Prognosis:1- and 3-year post-transplant mortality	19900 adults, UNOS dataset	Single split: 85%/15%	LAS: all pre-transplant recipient covariates; “clinician” model: 27 predictors; LASSO: 16 predictors; RF: 15 predictors	LAS, “clinician” model, LASSO and RF	AUC: 0.55–0.62 NPV: 0.87–0.90 PPV: 0.18–0.24 Specificity: 0.66–0.76 Sensitivity: 0.41–0.52	None reported
Dalton et al. (8)	Prognosis: 1-, 3- and 6-month waitlist survival, and 1-, 3-, and 5-year post-transplantation survival	13,204 candidates, 20,763 adult transplant recipients	10-fold cross-validation: split not reported	Socioeconomic and clinical factors	(I) WL-LAS/CAS model, (II) re-estimated WL-LAS/CAS model, (III) model II incorporating nonlinear relationships, (IV) RSF model, (V) logistic model, (VI) linear discriminant analysis, and (VII) GBT model	AUC: WL-CAS: 0.92 PT-CAS: 0.61	None reported
Sage et al. (9)	Prognosis: donor lung assessment	725 EVLP cases; single center	5-fold cross-validation: 80%/20%	Biological, physiological, and biochemical assessments	XGBoost, InsighTx model	AUROC: 79 ± 3% (training), 75 ± 4% (test dataset 1) 85 ± 3% (test dataset 2)	ΔpO2, static compliance, airway pressure, dynamic compliance, perfusate loss, base excess, pH, Ca2+, HCO3−, ΔpCO2
Pu et al. (10)	Diagnosis: predict donor's lung size	4610 subjects; single center	10-fold cross-validation: 80%/10%/10%	Chest CT scans and basic demographics	U-Net model, DT, RF, multiple linear regression, SVM, XGBoost, MLP, KNN, Bayesian regression	U-Net model: Dice coefficients Right lung: 0.951 ± 0.006 Left lung: 0.943 ± 0.007 Heart: 0.913 ± 0.035 Thoracic cavity: 0.959 ± 0.013 MLP (*R*^2^, mean absolute error, mean absolute percentage error): thoracic cavity volume: 0.628, 0.736 L, 10.9% right lung volume: 0.501, 0.383 L, 13.9% left lung volume: 0.507, 0.365 L, 15.2% XGBoost (*R*^2^, mean absolute error, mean absolute percentage error): total lung volume: 0.514, 0.728 L, 14.0% heart volume: 0.430, 0.075 L, 13.9%	None reported
Ram et al. (2)	Diagnosis: donor lungs assessment	100 subjects; a single-center prospective trial	Single split: 82.5%/17.5%	CT features, “ground truth”, clinically relevant parameters from both donors and recipients	Dictionary learning	None reported	None reported

In May 2005, the Organ Procurement and Transplantation Network in the United States implemented the lung allocation score (LAS) as a comprehensive system to prioritize lung transplant candidates. This scoring method incorporates a range of clinical metrics, including functional status, exercise tolerance, pulmonary function, hemodynamic parameters, and the requirement for supplemental oxygen or mechanical ventilation (11). The LAS employs a prediction model to assign candidates a normalized score between 0 and 100. Designed to balance medical urgency (waitlist mortality) with expected outcomes (one-year post-transplant survival), the LAS prioritizes candidates with higher scores, facilitating more equitable organ distribution. Since its introduction, LAS has successfully reduced waitlist mortality and increased LTx rates in the United States, along with initially a modest improvement in one-year survival post-transplant (12–14).

Still, there is potential for improvement. The comparatively low long-term survival of some LTx recipient subgroups, particularly older and younger patients, may not be adequately represented by one-year post-transplant survival. A more thorough assessment of the patients' long-term prognosis may be possible with an extended follow-up time (15, 16). Brahmbhatt et al. (7) evaluated the accuracy of different prediction models, including the LAS, least absolute shrinkage and selection operator (LASSO), random forests (RF) and “clinician” model, in predicting 1-year and 3-year post-transplant mortality in lung transplant patients. The study showed that both RF and “clinician” models improved short-term (1-year) prediction compared to LAS, but all models had low area under the curve (AUC) values (0.55–0.62). Although the negative predictive values (NPVs) were reasonable, ranging from 0.87 to 0.90, positive predictive values (PPVs) were low, all below 0.25. The LAS model calibration slope for 1-year post-transplant survival was 0.38 (95% CI [0.03, 0.73]), suggesting that the LAS overestimated mortality risk within this timeframe.

Additionally, all models' predictive performance was assessed by long-term (3-year) survival, fit by disease category, donor variables, and the LAS allocation era. However, none of the models improved performance for 3-year outcomes, with results generally worsening. The main limitations of the study included its reliance on data from the united network for organ sharing (UNOS) database provided by the Organ Procurement and Transplantation Network, which may not capture all relevant factors, potentially omitting key elements that influence long-term mortality outcomes. Furthermore, while the dataset was relatively recent, 3-year survival data from the final years remained limited, impacting the accuracy of long-term survival predictions. Given these limitations, applying short-term survival predictions from LAS and similar models based on pre-transplant recipient factors should be implemented with caution. For ethical organ allocation and efficient resource utilization, it remained crucial to develop accurate models to predict both medium- and long-term survival post-transplant.

Developing LTx risk-prediction models that integrate recipient, donor, and transplant characteristics to forecast long-term post-transplant survival is imperative. Zafar et al. (6) proposed an objective LTx allocation system that incorporates these factors by applying advanced statistical methods alongside ML and deep learning (DL) techniques. They employed a Cox-LASSO regression model to predict long-term survival probabilities and categorized recipients into three risk clusters (low, medium, and high) through the expectation-maximization clustering algorithm. Using these clusters, the researchers developed the Lung Transplantation Advanced Prediction Tool, a web-based tool to predict long-term survival probabilities at 1, 5, and 10 years, as well as the half-life for each recipient-donor match. The study, which involved a cohort of 19,263 eligible double LTx recipients, demonstrated good performance in predicting long-term survival probabilities across different risk groups. This study represents a significant first step towards developing more precise and dynamic risk prediction tools, such as the Advanced Prediction Tool, to assist in lung transplant decision-making and improve the accuracy of survival forecasts for transplant candidates.

LAS decisions constrained by strict geographic boundaries resulted in inequities, particularly disadvantaging patients residing near these boundaries who possess differing medical priorities. Such limitations can lead to lower-priority candidates receiving organs before higher-priority individuals outside the geographic zones (17).

To address these disparities, significant changes were introduced in March 2023 with the adoption of the composite allocation score (CAS), a new system for prioritizing lung transplant candidates (18). CAS promotes greater equity by removing traditional geographic constraints and prioritizing candidates based on a composite of geographic proximity and critical medical and socioeconomic factors (19).

To evaluate the effectiveness of the CAS model, Dalton and his team conducted a comprehensive study comparing its discriminative performance to alternative statistical and ML methods. The study also examined how socioeconomic and clinical factors influenced model performance (8). Researchers evaluated four models for predicting waiting list (WL) and post-transplant (PT) survival, including traditional WL-LAS/CAS models and their extended versions, as well as the random survival forests (RSF) model. In addition, survival stacking was employed with various ML techniques, such as logistic models and gradient boosting trees (GBT), to improve prediction accuracy. The study analyzed data from 13,735 candidates on the WL. WL models demonstrated high discriminative capability reflected by an AUC of 0.93, but performance declined in residual cohorts. For PT survival analysis, data from 20,763 adult transplant recipients were included. Compared to the WL models, the PT models exhibited lower and relatively stable discriminative performance, with AUC values ranging between 0.58 and 0.61. The performance slightly decreased over extended forecasting times and was worse in residual cohorts. Variability in WL and PT AUC was most pronounced among candidates on Medicaid, highlighting potential disparities tied to socioeconomic factors. Despite leveraging contemporary data and advanced modeling strategies, the study found no significant improvements in the discrimination performance of the CAS-based WL and PT survival models. This result indicates that current allocation models may have already achieved their maximum predictive power based on existing risk factors.

The CAS has yet to achieve universal acceptance worldwide, largely due to the lack of an internationally standardized model for lung donor-recipient allocation. Spanish researcher J. M. Dueñas-Jurado developed a novel allocation model informed by historical data from lung donors and recipients. This aims to optimize lung transplant outcomes and minimize morbidity and mortality in hospitals (5). The Logistic Regression using Initial Covariates and Product Units model is a hybrid framework combining classical statistical methods with newer ML approaches. It incorporates key variables influencing transplant survival, such as higher pre-transplant and post-transplant functional vital capacity, which have positive effects on survival with coefficients of 23.5 and 3.03, respectively. Lower forced expiratory volume in the first second (FEV1) negatively impacts survival with a coefficient of −23.51. While the model underscores promising factors, its reliance on single-center, retrospective data may limit generalizability. To bolster its credibility, further validation through cross-validation and longitudinal follow-up studies is necessary to improve predictive accuracy for long-term outcomes. Future efforts should focus on assessing its operational efficiency, clinical utility, and overall impact on patient care.

Increased risk for disease transmission (IRD) refers to organ donors who may transmit viruses such as hepatitis B virus, human immunodeficiency virus, and hepatitis C virus through transplantation (20, 21). Despite universal donor screening, there remains a risk of missed detection during the early infection “window period” when viral loads may be undetectable (4). As a result, over 19% of deceased organ donors are categorized as IRD for viral blood-borne disease transmission. Potential organ recipients must weigh the risks of accepting an IRD organ offer against the potential benefits of waiting for a non-IRD organ (21).

A study by Mark et al. (4) evaluated this complex decision-making process by developing transplant and waitlist survival models using RSF and the Cox proportional hazards model. These models simulated 20,000 scenarios comparing survival outcomes for patients accepting IRD organ offers vs. those waiting for non-IRD organs across heart, lung, and liver transplants. For lung transplants specifically, on average, recipients of IRD organs experienced a 7.2% higher 5-year survival probability than those who waited for non-IRD organs, despite an average wait time of 223 days. Notably, 69.9% of simulations favored IRD organ recipients, with the survival benefit increasing as waiting times grew. In scenarios with an estimated one-day wait, 49.4% of outcomes still indicated a higher survival chance for IRD organ recipients.

These findings underscore the potential of ML to refine organ allocation by improving survival predictions, optimizing donor-recipient matching, and reducing waitlist mortality. However, implementing such models in clinical practice requires addressing key challenges, including limited data acquisition, the need for generalizable models, and balancing short- and long-term survival predictions. By tackling these barriers, ML-based organ allocation can enhance the fairness and effectiveness of LTx.

### 2.2 Donor lung assessment

The success of LTx hinges on accurately assessing donor lung quality and ensuring size compatibility with the recipient. While computed tomography (CT) scans precisely evaluate recipient lung size, donor lung size and condition are often unavailable, with evaluations limited to basic demographic data. AI offers a promising solution to this gap. Pu et al. (10) utilized a U-Net model to segment lung, thoracic cavity, and heart structures from chest CT scans, calculating their respective volumes. Among eight ML methods tested on a cohort of 4,610 subjects, the multilayer perceptron (MLP) model was the best performer for predicting thoracic cavity and lung volumes, while the extreme gradient boosting (XGBoost) model excelled in heart volume predictions. These findings highlight AI's ability to estimate three-dimensional thoracic structure volumes using basic demographics, improving lung size matching for transplantation.

Traditional donor evaluations rely on donor history, clinical parameters, chest x-rays, bronchoscopy findings, and visual inspections by transplant surgeons (22–27). However, these criteria lack standardization and may exclude potentially viable lungs (28–30). The persistent gap between the number of patients on the lung transplant waitlist and the availability of suitable donor organs remains a critical challenge (31). To mitigate this, many programs have adopted extended donor criteria, increasing opportunities for patients with end-stage lung disease. With the increased use of suboptimal donor lungs, the development of objective techniques to support physicians in accurately screening donor lungs to identify recipients at the highest risk of post-transplant complications becomes particularly important.

Ram et al. (2) used ex vivo CT imaging combined with a supervised ML algorithm, “dictionary learning,” to screen donor lungs more effectively. This method leverages sparse representation-based classification to achieve high accuracy with limited datasets (32–34). In a study of 100 donor lung pairs, 70 were initially deemed suitable for transplantation through conventional clinical assessments prior to CT screening and then transplanted into recipients. Despite being screened, the remaining 30 pairs were not transplanted. ML algorithms successfully segmented 80 cases using a bespoke automated segmentation algorithm, of which 59 were accepted and 21 were declined for transplantation. Among the 52 donor lungs deemed suitable by clinical standards, the model predicted approximately 20% as unsuitable (“ML declined”), with these lungs demonstrating significantly lower feature probabilities (0.205 ± 0.042) compared to those accepted by the model (“ML accepted”) (0.637 ± 0.134, *p* < 0.0001). Recipients of ML-declined lungs faced poorer outcomes, including a median ICU stay of 14 days and a 19.13-fold increased risk (95% CI 3.98–91.80) of developing CLAD within two years post-transplant. The ML model showed 64% agreement with clinical decisions regarding the 14 non-transplanted donor lungs. The majority of ML-declined donor lungs had pulmonary complications, such as emphysema or pneumonia. This single-center study has some limitations: small sample size, model training and testing confined to a finite number of cases, and false-negative or false-positive risk. Therefore, the CT-ML strategy is not intended to replace the current donor lung screening system. Instead, it is designed to complement the existing screening process by integrating its algorithms, providing additional data support for clinical decision-making.

Ex vivo lung perfusion (EVLP) is a sophisticated medical technology that allows physicians to perform lung ventilation and circulatory perfusion in ex vivo, assessing and repairing donor lungs to ensure suitability for transplantation (35–40). This technology can increase the utilization rate of marginal donor lungs (41), offering a vital source of transplantable lungs for patients. However, traditional EVLP decision-making involves subjectivity, and the vast and complex clinical data produced during ex vivo perfusion—including physiological, biochemical, imaging, and biomarker measurements—can be particularly daunting for EVLP programs with less experience (42, 43). To establish a comprehensive approach for surgical decision-making using organ assessment data obtained during EVLP, Sage and colleagues developed InsighTx, an AI-driven decision-making tool based on the XGBoost algorithm (9). Leveraging clinical EVLP data from 725 cases collected at Toronto General Hospital (2008–2022), the dataset was split into training and testing datasets (80:20), enabling InsighTx to be trained to predict post-transplant outcomes. The InsighTx model the area under the receiver operating characteristic curve (AUROC) was 79 ± 3%, 75 ± 4%, and 85 ± 3% in training and independent test datasets, respectively. The model excelled in identifying transplants with good outcomes (AUROC: 80 ± 4%) and predicting unsuitable lungs for transplantation (AUROC: 90 ± 4%). In a retrospective blinded study, InsighTx influenced clinical decision-making, increasing the likelihood of transplanting suitable donor lungs (odds ratio = 13; 95% CI: 4–45) and reducing the likelihood of transplanting unsuitable donor lungs (odds ratio = 0.4; 95% CI: 0.16–0.98). These results have validated the safety and accuracy of integrating AI into EVLP protocols, supporting more precise decision-making and improving transplant outcomes. The development of InsighTx represents a milestone in advancing precision medicine in LTx. Future research will involve large external datasets and prospective, multi-center trials to confirm the study's conclusions. Additionally, exploring the expansion of biomarkers, implementing real-time monitoring, developing automated data extraction technologies, and integrating these with existing technological platforms will be key directions.

## 3 Post-transplant

### 3.1 Complications

Complications after LTx significantly impact patient outcomes in the short and long term (44). Given the complexity of these complications, standardized practices for identifying high-risk patients and implementing early interventions are currently lacking. ML technology can extract critical information from extensive and intricate biomedical data, supporting risk assessment, early diagnosis, and personalized treatment of complications after LTx (Table 2).

**Table 2:** Machine learning in post-transplant risk assessment and early diagnosis of chronic lung allograft dysfunction and other complications.

Reference	Objective	Dataset description	Dataset split (train/test)	Predictors	ML method/model	Evaluation of best model/Model performance	Top predictive variables
Weigt et al. (45)	Prognosis: CLAD	9 for incipient CLAD cases and 8 for CLAD-free controls; multicenter	Leave-one-out cross-validation	55 differentially expressed probe sets (map to 40 unique candidate genes)	SVM	Accuracy: 94.1%	Top 10 features
Barbosa et al. (46)	Diagnosis: BOS	176 LTx patients; single-center	10-fold jackknife split-sample cross-validation: 90%/10%	qCT, pulmonary function tests, semi-quantitative image scores, PCs, and qCT vol diff	Multiple variable linear regression and SVM	Unilateral LTx: SVM-utilizing PC from qCT: AUC = 0.817 Bilateral LTx: multiple variable linear regression-pulmonary function test: AUC = 0.765	Unilateral LTx: PC from qCT Bilateral LTx: pulmonary function test
Barbosa et al. (47)	Diagnosis: BOS	71 patients; multicenter	Cross-validation one-dimensional and two-dimensional spaces: 90%/10% three-dimensional space: 80%/20%	qCT functional respiratory imaging parameters	SVM	Accuracy: One-dimensional-76% two-dimensional-83% three-dimensional-85% Sensitivity: 73.3% Specificity: 92.3%	One-dimensional classification: central and total airway volumes at FRC Two-dimensional classification: right middle lobe volume at total lung capacity and right upper lobe volume at FRC Three-dimensional classification: Right middle lobe volume at total lung capacity, right upper lobe airway resistance at FRC, and central airway surface at FRC
Halloran et al. (48)	Diagnosis: lung transplant rejection	209 lung transplant recipients; 7 centers	Not applicable	Not applicable	Unsupervised ML	Not applicable	None reported
Halloran et al. (49)	Diagnosis: lung transplant rejection	214 patients; seven centers	Not applicable	Not applicable	Unsupervised ML	Not applicable	IFNG-inducible transcripts
Cantu et al. (50)	Diagnosis: predict recipient PGD risk	113 subjects; single center	5-fold cross-validation: split not reported	Clinical variables, toll-like receptor, and nod-like receptor signaling pathways	DL	Toll-like receptor signaling: AUC: 0.776 Sensitivity: 0.786 Specificity: 0.706 PPV: 0.471 NPV: 0.910	Toll-like receptor signaling
Halloran et al. (51)	Prognosis: establish the impact of molecular TCMR on graft survival	457 TBBs and 314 mucosal biopsies; 10 centers	Not applicable	Not applicable	Unsupervised ML	Not applicable	Molecular TCMR
Dugger et al. (52)	Prognosis: CLAD, new mechanistic biomarkers	22 CLAD cases and 27 matched controls; single center	Leave-one-out cross-validation	Gene expression data in airway brushes and TBBs	LASSO-penalized LR model, RF models	RF models-AUC TBBs: 0.62 (95% CI: 0.45–0.79) airway brushes: 0.84 (95% CI: 0.73–0.95)	Not all genes were explicitly listed
Berra et al. (53)	Prognosis: identification and progression of CLAD	40 lung transplant recipients; single center	Leave-one-out cross-validation	The concentrations of peptides from Ang II-regulated proteins in BAL fluid	SVM	AUC Discriminating between CLAD and no CLAD (stable + acute lung allograft dysfunction): 0.86 Predicting subsequent CLAD development: 0.97	Combinations of the seven peptides
Li et al. (54)	Diagnosis: predict recipients' PGD	113 enrolled subjects; single-center	5-fold cross-validation: split not reported	6 immunology pathways	PL	None reported	None reported
Stefanuto et al. (55)	Diagnosis: PGD biomarkers	35 lung transplant patients; single-center	Single split: 50%/50%	386 features	SVM	AUROC: 0.9 Accuracy: 0.83 Sensitivity: 0.63 Specificity: 0.94 PPV: 0.87 NPV: 0.80	Alkylated hydrocarbons, linear hydrocarbons, and aldehydes
Watzenboeck et al. (56)	Prognosis: predict future lung changes in lung function	19 patients; single center	Nested cross-validation	Clinical metadata, microbiome, metabolome and lipidome data sets	Ridge regression models	FEV1 (30 days): microbiome data (Pearson r = 0.76, *p* < 0.001) FEV1 (60 days): metabolome data + clinical metadata (Pearson r = 0.63, *p* < 0.001) FEV1 (90 days): clinical metadata (Pearson r = 0.42, *p* < 0.05)	FEV1 at 30, 60 or 90 days after sample collection
Su et al. (57)	Diagnosis: infection and rejection	59 lung transplant recipients; single-center	10-fold cross-validation: split not reported	6 bacterial genera, procalcitonin and T-lymphocyte levels	RF	AUC: Event-free vs. infection: 0.898; Event-free vs. Rejection: 0.919; Infection vs. rejection: 0.895	The combination of the 6-airway microbiota and PCT and T lymphocyte levels
McInnis et al. (58)	Prognosis: CLAD diagnosis for phenotyping and prognostication	88 patients; single center	Not applicable	NLML, HLML, GGOML, RETML, honeycombing, CTTLC, PVVML	The Computer-Aided Lung Informatics for Pathology Evaluation and Rating tool	Sensitivity: 0.90 Specificity: 0.71 Accuracy: 0.75 AUC: 0.851	Pulmonary vessel volume
Zhang et al. (59)	Diagnosis: transplant rejections	243 patients; gene expression omnibus database	10-fold cross-validation: split not reported	Number of genes: RF: 442, SVM: 247, KNN: 18, DT: 313	RF, SVM, KNN, DT	SVM: Accuracy: 0.992 Matthews correlation coefficient: 0.984	None reported
Wijbenga et al. (60)	Prognosis: CLAD, biomarkers	152 lung transplant recipients; single-center	10-fold internal cross-validation: 2:1	eNose sensor data and available known risk factors of CLAD	Supervised ML	Training set: AUC 0.94, sensitivity 96%, specificity 85%, accuracy 88% Validation set: AUC 0.94, sensitivity 100%, specificity 78%, accuracy 83%	eNose sensor data, available known risk factors of CLAD (age, gender, type of LTx, time after LTx and occurrence of any prior acute cellular rejection episodes)
Qin et al. (61)	Diagnosis: identification of cuproptosis-related biomarkers in allograft lung IRI	pre- and post-LTx lung biopsy samples and CRGs were obtained from the gene expression omnibus database and previous studies	Cross-validation Training set: 51 ischemic vs. 51 paired reperfusion (pre- vs. post-LTx) samples from GSE1,45,989 Validation set: 46 pre- vs. paired post-LTx samples from GSE127003	Fifteen differentially expressed cuproptosis-related genes	LASSO, SVM-RFE, RF	AUC: NFE2L2: 0.899 NLRP3: 0.874 LIPT1: 0.799 MTF1: 0.853	NFE2L2, NLRP3, LIPT1, and MTF1
Gouiaa et al. (62)	Diagnosis: acute cellular rejection	40 patients	5-fold cross-validation: 75%/25%	9 characteristics	Taelcore (MLP)	KL_0.01_: −138.563 KL_0.1_: −15.247 KL_1_: −0.208 Root mean square error: 0.0385 TRUST: 0.9464 Mean square error: 0.307	None reported
Gao et al. (63)	Diagnosis: explore NET-related gene biomarkers in IRI	Gene expression omnibus database	LASSO: 10-fold cross-validation; split not reported	Thirty-eight genes	LASSO and RF	AUC values exceeding 0.70 for all four genes	MMP9, PADI4, and S100A12
Michelson et al. (64)	Diagnosis: predict the development of PGD grade 3 within the first 72 h of transplantation	576 bilateral lung recipients; single-center	5-fold cross-validation: 75%/25%	11 variables	LR, KNN, XGB, and SVC	KNN: AUROC: 0.65 AUPRC: 0.45 F1: 0.62	Recipient sex-Female
Tian et al. (65)	Prognosis: AS	381 LTx patients; single center	Bootstrapping	15 variables	LR, DT, KNN, NB, SVM, GBRM, RF, XGB	RF AUC: 0.760 Brier score: 0.085 Sensitivity: 0.782 Specificity: 0.689 PPV: 0.252 NPV: 0.965	Postoperative 6-min walking test, diagnosis, sex, ECMO type, and preoperative hormone use

#### 3.1.1 Primary graft dysfunction

PGD, a severe form of acute lung injury that occurs within 72 h following LTx, remains a leading cause of early mortality (66–68). ML models may explore potential biomarkers, facilitate the early identification of risk factors, predict PGD development, and enable timely therapeutic interventions.

Ischemia-reperfusion injury (IRI) is a primary driver of early PGD, making exploring its pathogenesis a key research focus. Qin et al. (61) used three ML algorithms—LASSO, RF, and support vector machine recursive feature elimination (SVM-RFE)—to identify four key biomarkers from differentially expressed cuproptosis-related genes: LIPT1, NFE2L2, MTF1, and NLRP3, which are strongly associated with the pathogenesis of IRI. For each biomarker, the AUC of the ROC curve exceeded 0.8. Gene biomarkers associated with neutrophil extracellular traps in IRI during LTx were explored in another study (63). Using LASSO and RF algorithms, four candidate hub genes were screened out, with MMP9, PADI4, and S100A12 confirmed via enzyme-linked immunosorbent assay as upregulated post-reperfusion. These findings offer new perspectives on IRI and PGD potential therapeutic targets and early identification after LTx.

Cantu et al. (50) employed a feed-forward DL model to analyze gene expression data from donor lung tissue, with an emphasis on Nod-like receptor and Toll-like receptor signaling pathways, combined with clinical variables. The model showed excellent discrimination and precision in predicting recipient PGD risk. Peel learning (PL) for pathway-related outcome prediction is a specialized DL method that integrates the prior relationships among genes. It is particularly suited for gene expression studies with small sample sizes, high dimensionality, and strong inter-variable correlations. PL was applied in a case study by Li et al. (54) with a small cohort of lung transplant recipients, predicting PGD post-surgery using the donors' gene expressions within several immunological pathways. PL showed improved prediction accuracy compared to traditional DL methods. These combined gene expression and ML methods offer new possibilities for the early identification of PGD risk factors.

Michelson et al. (64) used the k-nearest neighbors (KNN) model, leveraging donor and recipient characteristics, to predict the onset of PGD grade 3 within the initial 72 h post-transplantation, with an AUROC of 0.65. Stefanuto et al. (55) applied the SVM algorithm to differentiate between severe- and lower-grade PGD by analyzing volatile organic compounds in blind bronchial aspirate samples and bronchoalveolar lavage (BAL) fluid collected within 6 h post-transplantation. The study revealed that severe PGD could be identified with an accuracy of 0.83 and an AUROC of 0.90. However, at later time points, the model's performance was moderate, achieving an AUROC of 0.80. These findings offer a scientific basis for the potential development of non-invasive, breath-based molecular diagnostic tools for assessing PGD.

#### 3.1.2 Airway complications

Airway complications are significantly associated with higher mortality after LTx (69). Airway stenosis (AS) is the most common complication, with incidence rates reported ranging from 1.6% to 32.0% in previous studies (70–74). Accurate and early detection of AS that requires therapeutic intervention, alongside enhanced early bronchoscopic surveillance post-LTx, may help to alleviate the disease burden. Tian et al. (65) developed an optimal ML model by analyzing the clinical profiles of 381 LTx recipients. Using the RF algorithm combined with feature selection based on the determination coefficient, the model can satisfactorily predict AS necessitating clinical intervention following LTx (AUC = 0.760, Brier score = 0.085). Five significant predictive features were identified, including the postoperative 6-min walking test, patient sex, diagnosis, type of extracorporeal membrane oxygenation (ECMO) employed, and preoperative hormone use.

### 3.2 Rejection

Poorly diagnosed rejection may result in excessive or insufficient immunosuppression (51). In transbronchial biopsies (TBBs), histologic evaluation of T-cell-mediated rejection (TCMR) is notably variable (75), often resulting in inconsistent diagnoses. Molecular diagnostics have emerged as an alternative to histology. The Molecular Microscope Diagnostic System is a proven tool developed to evaluate heart and kidney transplant biopsies with excellent technical reproducibility while using less tissue (76–79). It combines unsupervised ML-derived algorithms with microarray-based measurements. Halloran et al. (48) applied the centralized the Molecular Microscope Diagnostic System approach to diagnose molecular TCMR in single-piece TBBs with high surfactant transcripts. Mucosal biopsies, which are safer and easier to obtain than traditional TBBs, are also being explored. The molecular assessment of the third bronchial bifurcation has proven to be a powerful tool for evaluating lung transplant patients' disease status, capable of detecting rejection in previously unusable biopsy formats. This technique holds potential utility for patients with compromised respiratory function, where TBB is not feasible. The rejection phenotype identified in the third bronchial bifurcation is associated with IFNG-inducible transcripts, which are hallmarks of rejection (49). Additionally, Halloran and colleagues (51) conducted a study to train new ML models on larger datasets with the goal of identifying TCMR in all TBBs and investigating the correlation between graft loss risk and molecular TCMR. These models revealed that molecular TCMR, regardless of biopsy type, is a significant predictor of graft failure risk.

Advancements in technology are continuously enhancing the efficacy and precision of rejection monitoring after LTx. For instance, by analyzing gene expression profiling patterns of mucosal biopsies in conjunction with clinical outcomes from various studies, an SVM algorithm was able to distinguish patients into different rejection responses with a Matthews correlation coefficient of 0.984 and an overall accuracy of 0.992 (59). Additionally, Taelcore, a novel dimensionality reduction method, effectively reduces the dimensionality of high-dimensional datasets by integrating topological data analysis (80) and ML autoencoders (81), and excels at preserving the topological structure of the data. After being combined with MLP, the method achieved an accuracy of 90% in predicting the risk of acute cellular rejection after LTx (62). Su et al. (57) conducted 16S rRNA gene sequencing to analyze the airway microbiota from 181 sputum samples from 59 lung transplant recipients. The RF model, incorporating procalcitonin and T-lymphocyte levels, effectively discriminated between clinically stable recipients and those experiencing acute rejection and infection. These findings indicate that airway microbiota could serve as a potential biomarker to distinguish between acute rejection and infection following LTx, providing a non-invasive tool for post-LTx monitoring.

### 3.3 Chronic lung allograft dysfunction

CLAD remains a major cause of complications and mortality following LTx, affecting approximately 50% of recipients as early as four years post-transplant (82, 83). The primary phenotype of CLAD is bronchiolitis obliterans syndrome (BOS), which clinically manifests as physiologic airflow obstruction. Currently, there are no effective therapies that can prevent or reverse the confirmed CLAD (84), highlighting the importance of early detection to improve treatment outcomes.

Recent studies have demonstrated the potential of ML approaches for the early identification and risk stratification of CLAD and BOS, ultimately informing timely and personalized interventions in post-transplant care. As summarized in Table 2, several studies have explored diverse ML methodologies to enhance clinical decision-making. Two studies investigated the application of SVM in utilizing baseline quantitative CT metrics for the early diagnosis of BOS and predicting its eventual onset (46, 47). Both hierarchical clustering and SVM enable the correct classification of BAL fluid samples into CLAD-free and incipient CLAD categories (94.1% and 82.3%, respectively), supporting the potential of the transcriptome of BAL cell pellets as a biomarker for CLAD risk stratification (45). Another study further highlighted the value of SVM in distinguishing between CLAD from stable and acute lung allograft dysfunction patients at the time of bronchoscopy and predicting future CLAD development with precision (53). Additionally, RF models based on gene expression data from airway brushings outperformed traditional transbronchial biopsy samples, effectively distinguishing between CLAD and non-CLAD samples (52). McInnis et al. (58) used the Computer-Aided Lung Informatics for Pathology Evaluation and Rating tool, a validated ML algorithm for quantitatively analyzing lung texture on CT images automatically, to conclude that pulmonary vessel volume, a biomarker without direct visual correlation, is the strongest predictor for both CLAD phenotype classification and survival. Watzenboeck et al. (56) trained ridge regression models to predict short-term (30-day) changes in FEV1 after LTx using microbiome data. These insights are crucial for the early diagnosis of post-transplant lung function decline and timely intervention, which may prevent or delay the progression of CLAD. Wijbenga et al. (60) applied partial least squares discriminant analysis to reduce the dimensionality of electronic nose (eNose) sensor data, and evaluated its diagnostic value across CLAD phenotypes and stages. The eNose accurately discriminated between restrictive allograft syndrome and BOS. However, its discriminative ability was more limited for other CLAD phenotypes and CLAD stages.

### 3.4 Survival

Survival prediction is crucial in medicine, particularly amid organ shortages (85). Traditional statistical methods have several limitations, including assuming linear relationships between variables, relying on subjective variable selection, struggling with multicollinearity, and strict assumptions regarding data distribution (86–89). In contrast, ML can integrate diverse factors related to the recipient, donor, and transplantation process, providing a profound insight of the key influencing patient survival (90). Table 3 provides an overview of ML studies in survival prediction, detailing the types of ML tools evaluated and their performance metrics. Delen et al. (91) employed ML models combined with sensitivity analysis to identify a consolidated set of predictive variables, which were subsequently used to develop the Cox survival model for thoracic transplantation. Additionally, they applied the k-means clustering algorithm to classify patients into three risk groups, confirming significant differences among these groups through Kaplan–Meier survival analysis. Moro et al. (92) applied a survival tree algorithm to analyze recipient and donor data, identifying six key predictors: recipient age, post-transplant recipient ventilator and reintubation, duration of hospitalization from transplant to discharge, double LTx, and donor cytomegalovirus status. Tian et al. (93) found that the RSF model was more accurate than the Cox regression model in predicting overall survival for LTx patients. Based on variable importance, the final RSF model selected 16 factors, identifying postoperative ECMO duration as the most valuable variable. The RSF model demonstrated excellent performance, with an integrated Brier score of 0.130 (95% CI: 0.106–0.154) and an integrated area under the curve of 0.879 (95% CI: 0.832–0.921).

**Table 3:** Machine learning in patient and graft survival prediction.

Reference	Objective	Dataset description	Dataset split (train/test)	Predictors	ML method/model	Evaluation of best model/Model performance	Top predictive variables
Oztekin et al. (94)	Prognosis: predict the graft survival for heart-lung transplantation patients	16,604 cases; UNOS database	10-fold cross-validation: split not reported	283 variables	Prediction models (NN, DT, and LR) and Cox regression modeling	Accuracy NN: 79% to 86%; LR: 78% to 86%; DT: 71% to 79%	Sternotomy_Tcr; Angina_Cad; Pulm_Inf_Don; Func_Stat_Tcr; Death_Circum_Don; Age; Cig_Use
Delen et al. (91)	Prognosis: predict the survival time and determine risk groups of thoracic recipients	106,398 records; UNOS dataset	10-fold cross-validation: split not reported	372 cleansed independent variables and one dependent variable	SVM, ANN and DT	SVM: Mean square error: 0.023 *R*^2^: 0.879	14 significant variables with prognostic value
Amini et al. (95)	Prognosis: the impactful factors for a patient's lung transplant's prolonged survival	13,080 patients; UNOS dataset	10-fold cross-validation: split not reported	171 variables	GBT, RF, DT, KNN, ANN, SVM, LR	RF Accuracy: 77.92% Specificity: 79.58% Sensitivity: 76.26% AUC: 79%	Recipient CMV by IGG test results at transplantation
Jiao et al. (96)	Prognosis: identify the association of pulmonary artery pressure change during ECMO and post-LT survival	208 recipients; Chinese Lung Transplantation Registry	None reported	20 variables	XGboost	None reported	ΔmPAP (35 mmHg)
Tian et al. (93)	Prognosis: predict overall survival	504 patients; single center	Single split: 70%/30%	4 recipient factors, 1 donor factor, 4 transplant procedural factors, and 13 posttransplant factors	RSF	An integrated area under the curve: 0.879 (95% CI: 0.832–0.921) An integrated Brier score: 0.130 (95% CI: 0.106–0.154)	Postoperative ECMO time
Melnyk et al. (97)	Prognosis: identify the relationship between blood product transfusion and short-term morbidity and mortality following LTx	369 patients; single center	5-fold cross-validation: split not reported	Preoperative recipient characteristics, procedural variables, perioperative blood product transfusions, and donor characteristics	Elastic net regression	Average accuracy: 76.5% Sensitivity: 80% Specificity: 69% Balanced accuracy: 74%	11 significant predictors of composite morbidity, 3 protective predictors against composite morbidity
Chao et al. (98)	Prognosis: define and evaluate EVLP radiographic findings and their association with lung transplant outcomes	113 EVLP cases; single-center	Single split: 75%/25%	All radiographic lung scores from both time points and tabular EVLP parameters regularly taken clinically	XGBoost	AUROC: 80.7% ± 2.0%	First-hour consolidation and infiltrate lung scores
Moro et al. (92)	Prognosis: survival post-LT at 1, 5 and 10 years	27,296 lung transplant patients; UNOS dataset	10-fold cross-validation: 70%/30%	47 significant variables associated with mortality	Survival tree	C-index: 0.653	6 significant factors

Personalized and precise survival predictions are essential for guiding clinical decision-making and further improving postoperative survival in lung transplant patients. However, many ML models, particularly those using complex algorithms, are criticized for their “black-box”, where the decision-making processes remain opaque. Thus, Amini et al. (95) proposed an explanatory analytics framework to identify the key factors influencing long-term survival following LTx. Using the UNOS dataset, several ML algorithms were employed for classification, including GBT, RF, decision trees (DT), logistic regression (LR), SVM, KNN, and artificial neural networks (ANN). Among these, the RF model outperformed all others in almost all metrics (accuracy: 77.92%, specificity: 79.58%, sensitivity: 76.26%, and AUC: 79%). To provide interpretability for the best-performing model, the SHapley Additive exPlanations (SHAP) algorithm was used, revealing that FEV1 and Hepatitis B surface antibody were key predictors of long-term survival. Lung transplant researchers have not well examined these factors.

With respect to the impact of specific factors on LTx outcomes, Jiao et al. (96) used the XGBoost model to select and rank 20 variables, identifying pulmonary artery pressure change during ECMO as a key determinant of post-LT survival. Melnyk et al. (97) utilized elastic net regression to examine the effects of perioperative blood product transfusion, identifying 11 significant predictors associated with increased short-term morbidity and mortality following LTx. Chao et al. (98) focused on defining and evaluating EVLP radiographic findings, demonstrating that first-hour consolidation and infiltrate lung scores were predictive of transplant suitability and outcomes. Collectively, these studies underscore the importance of assessing and managing specific physiological and treatment-related factors to enhance LTx success and optimize patient outcomes.

ML models can also be employed to predict graft survival, which can better inform organ allocation and donor-recipient matching. Oztekin et al. (94) proposed an integrated data-mining methodology that utilized three different variable selection methods to identify a comprehensive set of factors, which were then employed to develop Cox regression models. The predictive models' performance ranged from 79% to 86% for NN, from 78% to 86% for LR, and from 71% to 79% for DT. This approach also revealed previously unknown patterns and relationships among the predictor variables.

### 3.5 Quality of life

Traditionally, most lung transplant referral guidelines have emphasized anticipated survival benefits. However, the primary motivation for patients to undergo LTx is the expectation of better health-related quality of life (99). LTx typically has a dramatic impact on a patient's health-related quality of life (100). Therefore, it is crucial to assess patient satisfaction regarding quality of life, considering multiple dimensions such as physical functioning, mental health, and social functioning for the postoperative clinical management of adult LTx recipients (101–103). Oztekin et al. (104) developed a hybrid feature selection methodology utilizing genetic algorithms (GA), aimed at achieving high classification accuracy of analytic models for predicting quality of life in LTx patients. They developed and successfully applied three decision analytic models—GA-SVM, GA-ANN, and GA-KNN—to address the feature selection. Among these, the GA-SVM model demonstrated superior performance. Furthermore, this study identified that recipient-cytomegalovirus IgG test results, simultaneous lung, and transplant type were key predictors of post-transplant health-related quality of life.

Although most LTx recipients experience improved lung function post-surgery, many may still encounter unexpected symptoms, such as dyspnea, exertional fatigue, and muscle pain. These symptoms can limit their ability to carry out daily activities and consequently have a negative impact on their overall quality of life (105, 106). In an observational cross-sectional study, Braccioni et al. (107) used a Forest-Tree ML approach to analyze the associations between symptom severity and cardiopulmonary exercise testing parameters. The study revealed several key correlations: the dyspnea score was significantly correlated with both minute ventilation at peak exercise and maximum power output; the muscle effort score was significantly correlated with breathing reserve as a percentage of maximal voluntary ventilation; and the muscle pain score showed significant correlations with oxygen uptake, arterial bicarbonate concentration at rest, and the minute ventilation/carbon dioxide production slope. These findings may inform the development of personalized symptom management and exercise rehabilitation programs for LTx recipients, ultimately improving their quality of life.

## 4 Discussion

**Figure 2:**
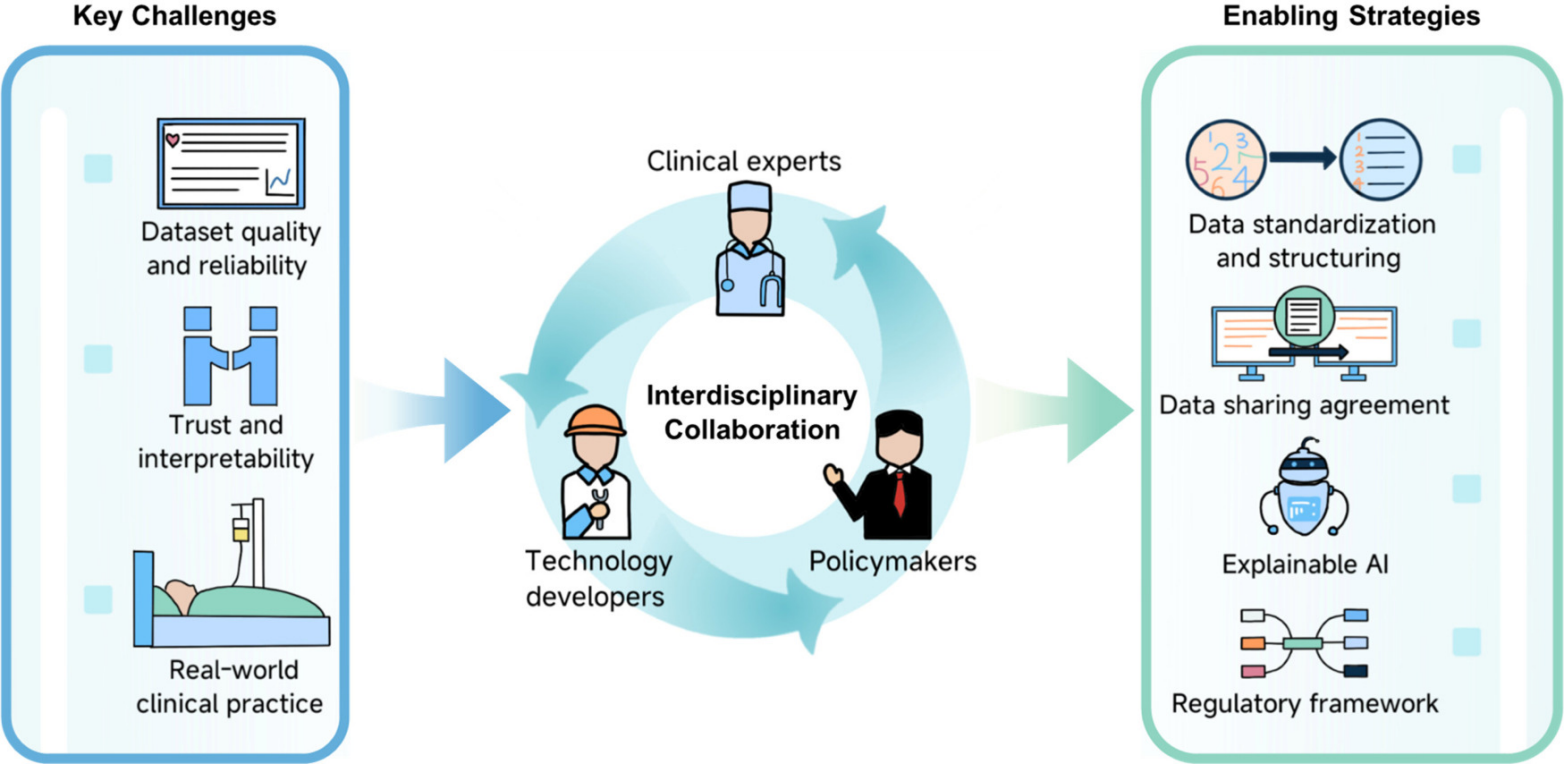
Enabling real-world AI in healthcare: challenges, strategies, and interdisciplinary collaboration.

Although numerous studies have demonstrated the immense potential of AI and ML in LTx, several challenges remain. Figure 2 provides a conceptual summary of the key barriers—related to dataset, model interpretability, and real-world implementation—and the potential strategies to address them, enabled by interdisciplinary collaboration.

### 4.1 Dataset quality and reliability

The performance of AI and ML models largely depends on access to large-scale, high-quality datasets (108). However, due to the need to protect patient privacy and ensure data security, data sharing among different medical institutions is restricted, which complicates the acquisition of sufficient post-LTx data, especially for rare complications or long-term follow-up data. Addressing these issues will require:

• Establishing cross-institutional data-sharing protocols and building a healthcare big data platform. Through techniques such as anonymization and encryption, data security and privacy can be preserved while simultaneously enhancing data sharing and quality control.
• Employing advanced ML techniques, such as oversampling or undersampling, or the development of new algorithms to handle imbalanced data (109).
• Adopting multi-center, prospective clinical trials to systematically and standardize data collection, along with rigorous data cleaning and validation processes.

These efforts are essential to improve data quality and reliability, facilitating the development of more accurate ML models.

### 4.2 Trust and interpretability

In ML, a “black-box model” refers to a model with an opaque relationship between input data and output results, characterized by complex and unexplainability algorithms (110, 111). In the medical field, where precise and reliable decision-making is essential, the opacity and unexplainability in AI models make it difficult for clinicians to understand the decision-making logic, thus undermining their trust in model predictions. Especially in auxiliary diagnosis requiring subjective judgment, this lack of trust may limit the model's applicability and acceptance in clinical practice. Furthermore, regulatory authorities may find it challenging to assess and monitor the performance and safety of these models, potentially leading to regulatory gaps and inadequacies, and raising ethical and legal concerns. Transparent model design must be prioritized to meet both clinical and regulatory expectations. To overcome these issues:

• Explainable AI such as SHAP can help clarify how specific features contribute to predictions, assisting clinicians in improving their decision-making processes (112).
• Case-Based Reasoning can use visual reasoning or visual explanations to help medical experts understand why cases are similar by visualizing shared patient characteristics, thereby enhancing the acceptance and applicability of AI models in clinical practice (113).

In medical AI systems, clinical validation is the most critical requirement for translating algorithmic outputs into real-world decision-making.

### 4.3 Real-world clinical practice

The global scarcity of medical resources makes the demand for AI applications in healthcare widespread and urgent. The efficient computational capabilities of AI can assist physicians in diagnosis and decision-making, allocate medical resources rationally, and promote the personalization and precision of healthcare services (114). AI systems must undergo broader testing and prospective evaluation in real-world clinical practice to ensure their accuracy, safety, and practicality (115). Successful integration of AI models into clinical workflows requires overcoming sociotechnical challenges, including:

• Training and educating users—including physicians and patients—to ensure they can effectively utilize AI and have confidence in its decisions.
• Continuously monitoring AI systems' emerging behaviors and user responses in complex sociotechnical environments after the deployment (116).
• Establishing a robust governance structure and regulatory framework to ensure the safety and efficacy of AI, addressing issues of transparency, responsibility, accountability, as well as clear guidelines regarding data ownership and usage rights.

Through these comprehensive measures, we can better harness AI technology to enhance the quality and accessibility of healthcare services.

## 5 Conclusion

In summary, the application of AI and ML in the field of LTx encompasses several key aspects, from pre-transplant organ allocation and donor assessment to post-transplant clinical outcome prediction and diagnosis of postoperative complications. These technologies can analyze and understand the complex relationships among variables, enhancing prediction accuracy. Existing research has demonstrated the potential of ML in clinical applications for LTx, but it remains in the early stages of development. Currently, several barriers persist, including insufficient standardization and structuring of data, a lack of multicenter prospective clinical trials, physicians' distrust of ML, and ethical and regulatory issues. Addressing these challenges requires interdisciplinary collaboration among clinical experts, technology developers, and policymakers to facilitate the successful deployment and scaling of AI and ML in the healthcare sector.
